# New Nuclear SNP Markers Unravel the Genetic Structure and Effective Population Size of Albacore Tuna (*Thunnus alalunga*)

**DOI:** 10.1371/journal.pone.0128247

**Published:** 2015-06-19

**Authors:** Urtzi Laconcha, Mikel Iriondo, Haritz Arrizabalaga, Carmen Manzano, Pablo Markaide, Iratxe Montes, Iratxe Zarraonaindia, Igor Velado, Eider Bilbao, Nicolas Goñi, Josu Santiago, Andrés Domingo, Saadet Karakulak, Işık Oray, Andone Estonba

**Affiliations:** 1 AZTI, Marine Research Division, Herrera Kaia Portualdea z/g, Pasaia (Gipuzkoa), Spain; 2 Department of Genetics, Physical Anthropology and Animal Physiology, University of the Basque Country (UPV/EHU), Sarriena auzoa z/g, Leioa (Bizkaia), Spain; 3 Cell Biology & Environmental Toxicology Research Group, Research Centre for Experimental Marine Biology & Biotechnology (PIE) & Department of Zoology & Animal Cell Biology, University of the Basque Country (UPV/EHU), Sarriena auzoa z/g, Leioa (Bizkaia), Spain; 4 AZTI, Marine Research Division, Txatxarramendi Ugartea z/g, Sukarrieta (Bizkaia), Spain; 5 Laboratory of Pelagic Resources, Dirección Nacional de Recursos Acuáticos (DINARA), Constituyente 1497, Montevideo, Uruguay; 6 Fisheries Faculty of Istanbul University, Laleli, Istanbul, Turkey; Tuscia University, ITALY

## Abstract

In the present study we have investigated the population genetic structure of albacore (*Thunnus alalunga*, Bonnaterre 1788) and assessed the loss of genetic diversity, likely due to overfishing, of albacore population in the North Atlantic Ocean. For this purpose, 1,331 individuals from 26 worldwide locations were analyzed by genotyping 75 novel nuclear SNPs. Our results indicated the existence of four genetically homogeneous populations delimited within the Mediterranean Sea, the Atlantic Ocean, the Indian Ocean and the Pacific Ocean. Current definition of stocks allows the sustainable management of albacore since no stock includes more than one genetic entity. In addition, *short*- and *long-term* effective population sizes were estimated for the North Atlantic Ocean albacore population, and results showed no historical decline for this population. Therefore, the genetic diversity and, consequently, the adaptive potential of this population have not been significantly affected by overfishing.

## Introduction

Albacore tuna (*Thunnus alalunga*, Bonnaterre 1788) is distributed in the Atlantic, Pacific and Indian Oceans and in the Mediterranean Sea, extending from 50–55°N to 40–45°S [1]. This species is the fourth most important one of the *Thunnus* genus with regard to captures [2]. This fact reflects the high commercial value of the albacore and its related products, which makes this species likely to be exploited beyond its maximum sustainable yield [1]. Migrations on this species has been studied for several decades through tag-recapture experiments showing low rate of albacore migration between hemispheres [3], and no transoceanic [3,4,5] neither Atlantic-Mediterranean migrations [6]. There are very few studies on spawning areas of this species, because catching larvae or young-of-the-year individuals (reference samples) of this species is not a very common event. One spawning ground has been defined in the western Mediterranean [7–9], two spawning areas in the North Atlantic Ocean [10,11], a single one in the South Atlantic [10], one spawning area in the Indian Ocean [12,13], and two Pacific separate spawning grounds: north and south [14–16]. According to this knowledge on population dynamics of albacore, six stocks or management units are currently defined by Regional Fisheries Management Organizations (RFMOs): (i) Mediterranean Sea, (ii) North Atlantic Ocean, (iii) South Atlantic Ocean, (iv) Indian Ocean, (v) North Pacific Ocean and (vi) South Pacific Ocean. Many fisheries are regulated in accordance with spatial schemes. However, management units based only on knowledge about migrations do not necessarily correspond to the biological structure of the populations [17,18]. In these cases, when fishery management is not based or does not fit the biological structure, changes may occur in the biological attributes, productivity and genetic diversity of the exploited species [19]. Therefore, the establishment of an accurate population boundary for a commercial species requires a multidisciplinary approach, and genetic studies can contribute very valuable information in this regard [20,21]. Thus, studies including population genetic structure assessment together with other population identification methodologies, such as tag-recapture [6] or chemical tags in otoliths [22], have become more common in the last decade. These multidisciplinary studies allow a more accurate population structure and hence, more sustainable fisheries management policies.

A variety of studies have assessed population structure of albacore species using multiple approaches including: otolith microstructure [23,24], tag-recapture methods [6], morphometrics [25] and genetic techniques [26–34]. The population structure of albacore has been found to exhibit a high dispersal capacity (e.g. [35]), similarly to what happens to other marine species such as Atlantic bluefin tuna (*Thunnus thynnus*) [34,36] or Atlantic mackerel (*Scomber scombrus*) [37]. However, despite the number of studies performed since the last decade, genetic structure of albacore is not clear yet, since contradictory information about number of albacore populations and population boundaries have been reported. In this regard, Albaina et al. [34] suggested four albacore populations (one in each ocean and one in the Mediterranean Sea), but Pujolar et al. [38] and Graves and Dizon [27] found genetic homogeneity between the Atlantic Ocean and Mediterranean Sea and Atlantic and Pacific Oceans, respectively, and Montes et al. [33] found homogeneity between the Atlantic and Indian Oceans. Moreover, genetic structure within oceans remains unclear since heterogeneity within them or within the Mediterranean Sea has been suggested [6,26,29,32,33,39]. Comparison between studies is difficult because differences on genetic markers studied and also on geographic areas assessed, which in certain studies are very limited into the bargain. In fact, few studies have addressed the population structure of albacore covering the worldwide distribution range of the species [1,33,34].

The North Atlantic albacore tuna stock was subjected to overfishing conditions between the mid 1960s and mid 2000s. As a result, the spawning stock biomass had been overexploited (below levels associated to the maximum sustainable yield) since the 1980s, but is now recovering over the last decade [40].After the population genetic structure of a species is defined, an essential parameter that informs about the sustainable management and conservation of exploited species is the effective population size (N_e_) [41]. While population genetic structure enables a definition of populations, that can be linked to the stock or management unit concept, N_e_ determines how vulnerable these populations are to losing genetic diversity due to genetic drift [42] and consequently, this variable assesses their responsiveness and adaptation capabilities. Despite the importance of this parameter for populations’ conservation, few studies have estimated N_e_ for tunas [36,43,44,45,46].

In summary, a number of outstanding issues persist which have direct implications for the sustainable management of albacore. These main questions to be answered include (1) the absence of a consensus about the genetic structure of this species worldwide, and (2) uncertainty about the impact of fishing on the effective population size (N_e_) and, therefore, on the genetic diversity of albacore populations. The goal of this study is to obtain a clear definition of the population genetic structure of albacore, and to shed light on its genetic viability via the estimation of N_e_ for the North Atlantic population, with the aim of providing a more rational foundation for sustainable fishery management. With this objective in mind, we carried out the most extensive sampling of albacore to date, covering its worldwide distribution range (1,331 samples from 26 locations worldwide). The number of markers employed was also the highest used to date, involving 115 novel nuclear SNP markers which we report in albacore tuna through cross-species transcriptome amplification and sequencing.

## Material and Methods

### Samples and DNA extraction

**Fig 1:**
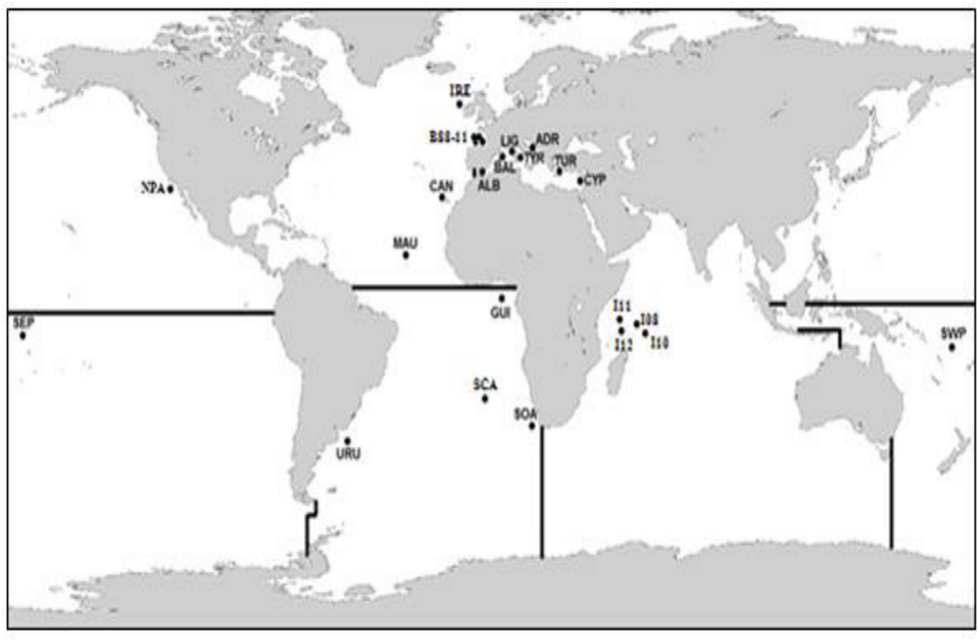
Sampling locations. Current stock boundaries delineated with black lines. Sample abbreviations are as defined in Table 1.

An exhaustive spatial-temporal sample of 1,331 albacore individuals from 26 locations covering the whole geographical distribution of the species was obtained (Fig 1, Table 1). The total sample includes 774 individuals from the Atlantic Ocean (12 locations sampled over 24 years), 254 individuals from Mediterranean Sea (7 locations sampled over 12 years), 136 individuals from the Indian Ocean (4 locations within 4 years of sampling), and 167 individuals from the Pacific Ocean (3 locations sampled over 5 years). Individuals were mainly sampled between 2008 and 2012, with some individuals sampled in previous years as far back as 1988. Sampled individuals were provided either by commercial or recreational vessels or by oceanographic institutes that collected the samples during scientific surveys. All fish were collected as part of authorized routine fishing procedures and therefore did not require any special additional permission. Some samples were used in previous studies [6,32–34,47] (Table 1). Collected tissues mainly consisted of muscle, fin or heart tissue, and they were stored either frozen at -20°C or preserved in 96% ethanol at 4°C. Additionally, spine cuts mounted in Eukitt (O. Kindler GmbH), as well as dried and stained blood samples were collected (Table 1). DNA from muscle, fin and heart tissue samples was extracted using NucleoSpin 96 Tissue Kit (Macherey-Nagel). Spine and blood samples were first immersed in xylol, and spine samples were afterwards manually crushed; DNA from these samples was extracted by means of a specific membrane using QIAmp DNA Investigator Kit (Qiagen). DNA from all samples was quantified using both a NanoDrop 1000 spectrophotometer (Thermo Fisher Scientific Inc., Waltham, MA) and a Qubit 2.0 (Invitrogen, Life Technologies) fluorometer. All DNA samples were stored at -20°C for subsequent analyses.

**Table 1:** Sample number (#), name, location, year of capture, number of individuals (N), stock, geographic coordinates and sample type (T, muscle, fin or heart tissue; B, blood; S, spine).

#	Name	Location	Year	N	Stock	Latitude	Longitude	Sample type
1 ^A^	ADR	Adriatic Sea	2006	48	Mediterranean Sea	41.29	17.52	T
2 ^A^ ^,^ ^B^ ^,^ ^C^	BAL	Balearic Sea	2005	31	Mediterranean Sea	40.00	1.58	T
3	CYP	Cyprus	2011	10	Mediterranean Sea	36.08	33.68	T
4	TUR	Turkey	2011	53	Mediterranean Sea	35.04	26.80	T
5 ^A^	TYR	Tyrrhenian Sea	2008	48	Mediterranean Sea	38.88	11.74	T
6	LIG	Ligurian Sea	2011	27	Mediterranean Sea	43.38	9.05	T
7^D^	ALB	Alboran Sea	1999	37	Mediterranean Sea	36.23	-2.00	B
8 ^E^	B88	Bay of Biscay	1988	34	North Atlantic Ocean	45.10	-4.35	S
9 ^E^	B89	Bay of Biscay	1989	30	North Atlantic Ocean	45.64	-4.76	S
10 ^A^ ^,^ ^B^	B09	Bay of Biscay	2009	42	North Atlantic Ocean	45.05	-5.28	T
11	B10	Bay of Biscay	2010	240	North Atlantic Ocean	45.71	-5.53	T
12	B11	Bay of Biscay	2011	31	North Atlantic Ocean	44.92	-4.16	T
13	CAN	Canary Islands	2012	41	North Atlantic Ocean	27.73	-17.25	T
14 ^A^ ^,^ ^B^	IRE	Ireland	2008	57	North Atlantic Ocean	54.17	-12.89	T
15	MAU	Mauritania	2010	48	North Atlantic Ocean	9.79	-32.16	T
16	GUI	Gulf of Guinea	1999–2000	32	South Atlantic Ocean	1.98	-16.58	B
17	URU	Uruguay	2005, 2007–2012	84	South Atlantic Ocean	-36.19	-53.16	T
18 ^A^ ^,^ ^B^	SCA	South Africa	2009	98	South Atlantic Ocean	-24.56	4.42	T
19	SOA	South Africa	2011	37	South Atlantic Ocean	-34.34	18.00	T
20 ^A^ ^,^ ^B^	I08	Seychelles	2008–2009	23	Indian Ocean	-7.11	54.65	T
21	I10	Seychelles	2010	38	Indian Ocean	-7.27	56.32	T
22	I11	Seychelles	2011	42	Indian Ocean	-7.28	49.06	T
23	I12	Seychelles	2012	33	Indian Ocean	-8.86	49.13	T
24 ^A^ ^,^ ^B^	NPA	California	2008	83	North Pacific Ocean	43.50	-127.00	T
25 ^A^ ^,^ ^B^	SEP	New Caledonia	2004–2005	51	South Pacific Ocean	-19.01	-152.84	T
26 ^A^ ^,^ ^B^	SWP	French Polynesia	2003–2008	33	South Pacific Ocean	-18.53	165.97	T
	** **		1988–2012	Total = 1,331				

^A^ Sample previously analyzed in [33]

^B^ Sample previously analyzed in [34]

^C^ Sample previously analyzed in [32]

^D^ Sample previously analyzed in [6]

^E^ Sample previously analyzed in [47]

### SNP selection and genotyping

The SNPs used in this study were previously discovered in the closely related ABFT species through transcriptome and genome sequencing, using 454 (GS FLEX Titanium) and HiSeq2000 (Illumina), respectively (Cariani et al. Personal Communication). Of all the discovered SNPs in the ABFT species, 384 transcriptome SNPs were genotyped using the GoldenGate platform (VeraCode), in 30 albacore samples covering the entire distribution range of the species (5 individuals from each defined management unit: North Atlantic, South Atlantic, Mediterranean Sea, Indian Ocean, North Pacific and South Pacific). From the 384 ABFT SNPs, only those that successfully amplified in albacore (conversion rate) and have Minor Allele Frequency (MAF) values above 0.01 in the latter species were taken into account. From these, only those markers that were compatible with TaqMan OpenArray technology (Life Technologies) were selected for this study. Additionally, two nuclear SNPs, previously described for albacore ([34]; S1 Table), were included in the final SNP set as a positive control, in order to corroborate the correctness of the genotyping procedure. Thus, a final set of 117 SNPs was designed to genotype 1,331 albacore individuals through TaqMan OpenArray technology. Validation rate was calculated as the proportion of SNP with a MAF > 0.001. In order to ensure genotyping quality, SNPs needed to comply with the following criteria: a call rate higher than 80%, clear genotyping clusters, and compliance with Hardy-Weinberg equilibrium (HWE).

### Statistical analysis

GENEPOP v4.0 [48] software was used to test departures from HWE and to analyze genotypic disequilibrium (GD) between SNPs (*p*-value < 0.001). Linked SNPs were phased into haplotypes using PHASE v2.1 software [49].

In order to assess the genetic population structure of the albacore, Reynolds genetic distance matrices [50] were obtained using Populations v1.2.32 software [51]. A Neighbor-Net dendrogram was constructed using SPLITSTREE v4.13 [52] based on the matrix of genetic distances. Geographic distance was calculated measuring the shortest distance by sea between each pair of sample location using scripts from the Movable Type Ltd webpage (http://www.movable-type.co.uk/scripts/latlong.html). Isolation by distance (IBD) was tested evaluating the correlation between Rousset's genetic distance [53] and geographic distance, using Mantel test implemented in IBDWS [54] with 30,000 randomizations. Population genetic structure was also assessed using STRUCTURE v2.3.4 [55] and GENELAND v3.2.2 [56] software, which are based on Bayesian clustering algorithms that allow assigning individuals to a group without previous assumption of either population units or population boundaries. STRUCTURE was run using the mixed ancestry model and correlated allele frequencies [57], using information regarding sampling location. Ten independent runs were simulated for each potential number of populations (K) with values of K = 1–6, and with a burn-in period of 50,000 Markov chain Monte Carlo (MCMC) steps, followed by 500,000 MCMC steps. The best K was estimated as proposed by Pritchard et al. [55]. CLUMPP v1.1.2 [58] was used to determine the optimal assignation of clusters for the analyzed individuals, maximizing similarity between the 10 different STRUCTURE replications for the selected K. Individual membership coefficients were graphically shaped with DISTRUCT v1.1 [59]. Finally, to test potential weaker structure within the detected major clusters, STRUCTURE analysis was repeated for each of them. While STRUCTURE is based only on the individual genotype data to infer the population structure, GENELAND uses the geographical information of the individuals as an additional parameter in the analysis. In the latter case, K was estimated from 1 to 5, using 500,000 MCMC iterations and 1,000 thinnings. Ten runs with fixed K were then post processed using a burn-in of 50,000 iterations to obtain the posterior probabilities of population membership for each individual and each pixel of the spatial domain.

We searched for candidate *loci* under selection (outlier *loci*) using the Bayesian likelihood method, as implemented in BAYESCAN v2.1 [60], with 10 pilot runs of 5,000 iterations and an additional burn-in of 50,000 iterations (sample size of 5,000 and thinning interval of 10). Critical values for the test were adjusted with false discovery rate (FDR) procedure (*q*-value < 0.05) [61]. Pairwise F_ST_ [62] values among samples based on neutral markers were estimated with FSTAT v2.9.3 software [63]. P-values were weighted using the FDR method for multiple testing [61].

The statistical power required to detect various levels of differentiation with the SNPs used in this study was estimated using POWSIM version 4.1 [64]. Since POWSIM is restricted to 50 loci, we selected those 50 loci with highest F_ST_ values. Burn-in consisted of 1000 steps followed by 100 batches of 1000 steps. Chi-square probabilities were used to test the significance of an F_ST_ value for each replicate run. The number of significant F_ST_ values in 1000 replicate simulations provided an estimate of the statistical power for a given level of divergence, which was controlled by allowing frequencies to drift for a given number of generations. Simulated effective populations sizes equaled 2000 fish.

Two different time-scale N_e_ estimates were obtained for the North Atlantic stock. *Short-term* N_e_ was estimated from temporal fluctuations in allele frequencies between cohorts [65], and a correction for overlapping generations was applied [66–68]. Generation time (Ĝ) was estimated following Felsenstein [69] from age frequency data of analyzed years (1988–2012), and changes in allele frequencies among cohorts were measured by F_S_ [67]. The *long‐term* N_e_ [70] uses a maximum likelihood estimator based on the coalescence theory. It is a retrospective model of population genetics which traces back for the most recent individual from which all organisms in a group are directly descended, the most recent common ancestor (MRCA). This tool has been employed to estimate historic population sizes for a range of species [71].

**Fig 2:**
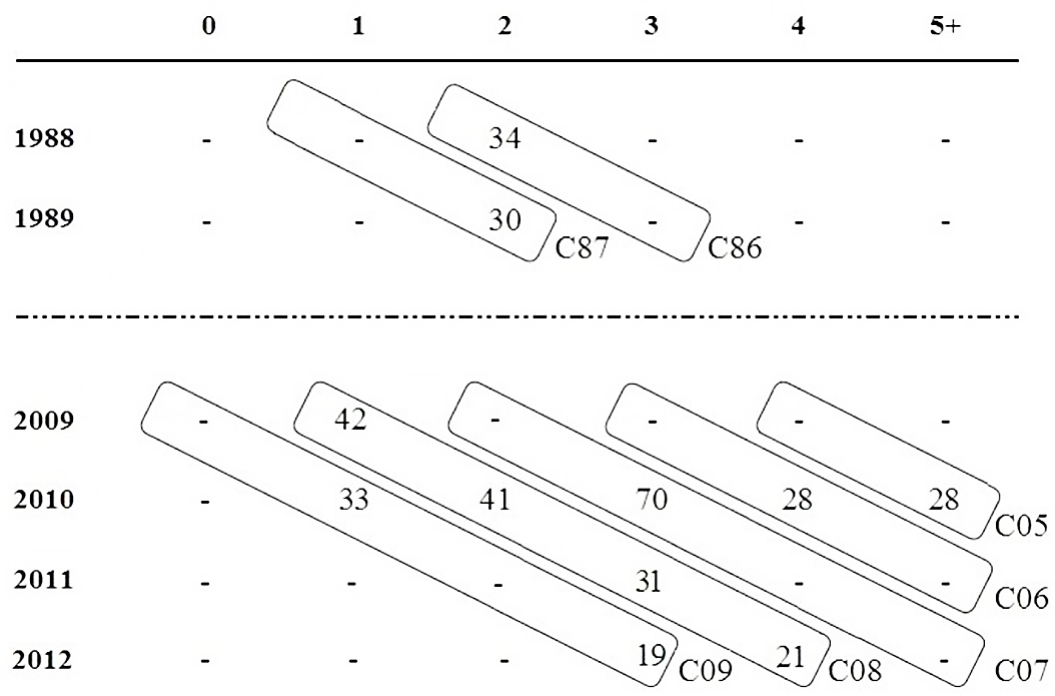
Diagram of the defined cohorts, based on the age of the individuals. Columns indicate age of individuals and rows year of capture. Values inside tables are the number of individuals for each age/year combination. Seven cohorts were defined by diagonal frames whose names were based on the hatching year of the individuals: C86, C87, C05, C06, C07, C08 and C09.

Data for North Atlantic albacore were obtained between 1988–2012, which constitutes 4–5 generations of albacore assuming 50% maturity at age 5 [25]. Age was estimated using length and weight information according to Santiago [72] and Santiago and Arrizabalaga [47]. We used age-structure data for seven cohorts (Fig 2). Cohort analysis was carried out to assess temporal fluctuations in population size. The adult population size (N_c_) in the North Atlantic, obtained from the report of the 2013 ICCAT North and South Atlantic albacore stock assessment [40], was compared with total population size N_e_ estimates. MIGRATE v3.2.1 software [73] was used for *long‐term* N_e_ estimation, and mutation was modeled by an infinite allele model.

## Results

### SNP selection and genotyping

From the 384 ABFT SNPs analyzed in the 30 albacore sample, 311 SNPs (conversion rate = 80.99%) successfully amplified in albacore, and among them, 121 showed MAF > 0.01 (31.51%). From these, 115 SNPs exhibited compatibility with the TaqMan OpenArray technology (Life Technologies), and were further genotyped together with the 2 nuclear SNPs included as a positive control.

Out of 117 nuclear SNPs, 95 were polymorphic (they had a MAF value above 0.001, i.e. the minor allele was observed at least 5 times) and had a clear genotype for the 1,331 albacore individuals (S1 Table). Therefore, validation rate was 24.61% (95/386). From these, 76 met HWE. The exact tests for genotypic disequilibrium (GD) detected 2 SNPs (ss974292126 and ss974292127) with significant GD probabilities, so these 2 SNPs were phased into one haplotype (ss974292126+ss974292127). Therefore, a set of 75 independent nuclear markers was downstream analyzed.

### Population structure

**Fig 3:**
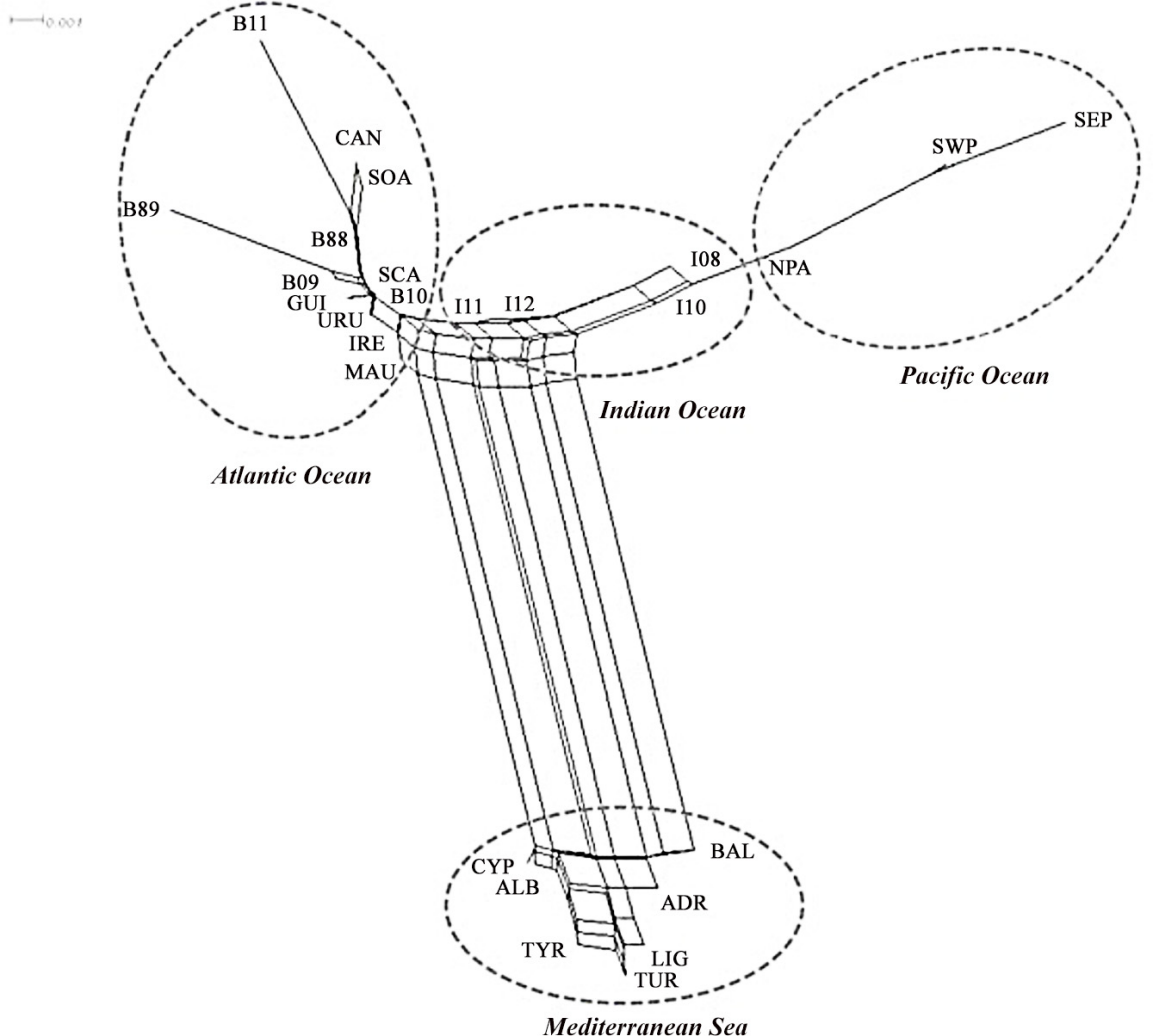
Neighbor-Net dendrogram built from Reynolds distances between 26 samples. Sample abbreviations are as defined in Table 1.

The Neighbor-Net drawn from Reynolds genetic distances (Fig 3) grouped locations according to their geographical region. The Mediterranean Sea samples, grouped into a single cluster, were the most distant from the rest. The samples from the three oceans also grouped by ocean, and those from the Indian Ocean were placed between those of the Atlantic and those of the Pacific. The genetic and geographic distances for the 26 samples showed a significant correlation (r = 0.4577, p < 0.0001; S1 Fig). This correlation increased notably when the Mediterranean samples were removed from the analysis (r = 0.7549, p < 0.0001; 19 locations). Within the Mediterranean, no significant correlation was found between genetic and geographic distances (r = -0.3210, p = 0.0954; S1 Fig).

**Fig 4:**
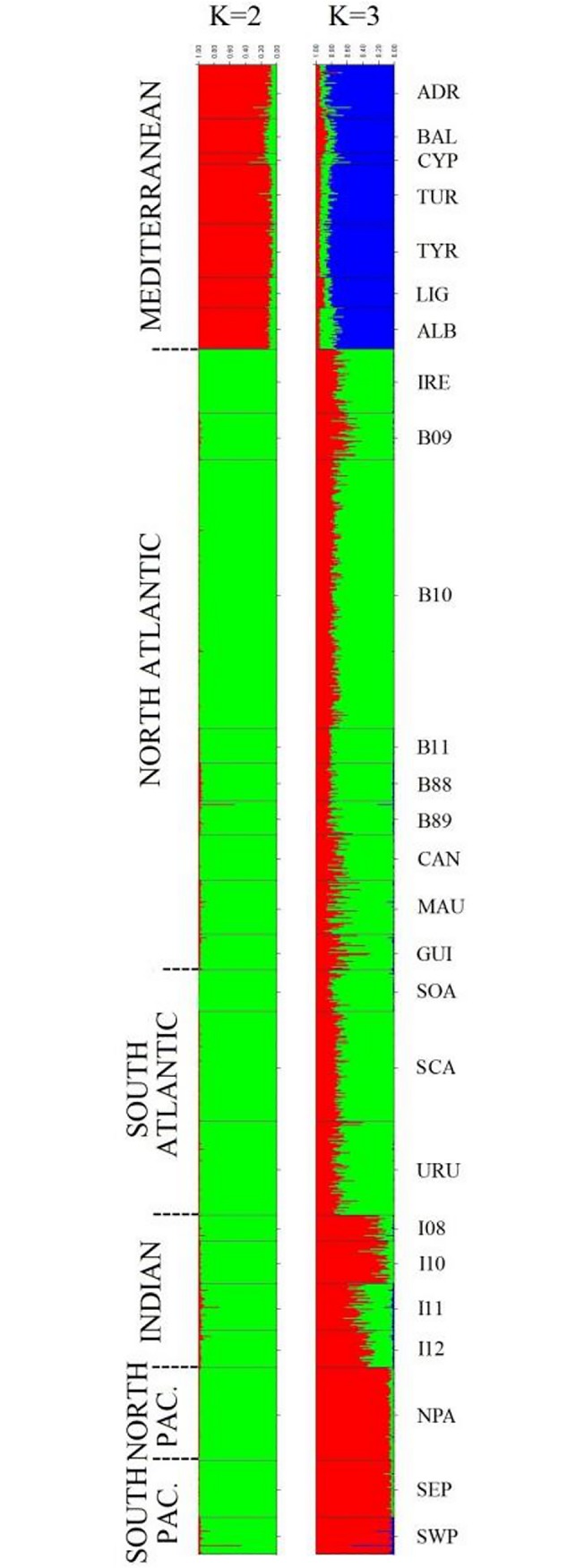
Individual clustering analysis implemented with STRUCTURE software for K = 2 and K = 3. Each vertical bar represents an individual. The 26 locations are separated by horizontal continuous black lines, and the currently accepted 6 stocks are separated by discontinuous horizontal black lines. The color proportions of each bar correspond to individuals’ estimated membership fractions of each of the clusters. Sample abbreviations are as defined in Table 1.

With respect to the analysis of individual clustering using the STRUCTURE software, when 2 group clusters were considered (K = 2) a clear distinction could be observed between the samples from the Mediterranean Sea and the others (Fig 4). In any event, the best K value obtained was 3 (S2A Fig), which clearly distinguished the Pacific Ocean (red) samples from those of the Atlantic Ocean (mostly green; Fig 4). The case of samples from the Indian Ocean is special in that we observed intermediate percentages of the components of the Atlantic and the Pacific. In the same way, the GENELAND software also detected K = 3 as the most probable number of groups (S2B Fig). In this analysis, the 3 clusters were made up of the Mediterranean samples (cluster 1), the Atlantic samples (cluster 2) and the Indo-Pacific samples (cluster 3). When STRUCTURE analysis was repeated for each of the major clusters, no structure was detected within them, since the best K value obtained was 1 for all the analysis.

A total of 17 out of the 75 independent markers were identified as outliers, therefore, 58 SNPs were defined as neutral SNPs. Heterogeneity analyses performed within stocks based on the 58 neutral SNPs revealed that the 6 stocks defined by the Regional Fisheries Management Organizations (RFMOs) were genetically homogeneous (p > 0.05; Table 2). POWSIM simulations showed that the 50 SNPs with the highest F_ST_ values together were able to detect significant differences among samples with F_ST_ = 0.0015 in about 95% of the tests, and with F_ST_ = 0.002 in 100% of the tests (Table 3). The F_ST_ values between stocks varied from a minimum F_ST_ = 0.001 between the North and South Atlantic and between the North and South Pacific, and a maximum F_ST_ = 0.051 between the South Pacific and the Mediterranean stock. All comparisons were found to be statistically significant, except those obtained between the North and South Atlantic, the North and South Pacific and between the North Pacific and the Indian Ocean (Table 2).

**Table 2:** Pairwise F_ST_ values (below the diagonal) and *p*-values (above the diagonal) between the 6 stocks currently recognized by the RFMOs.

	MED	NATL	SATL	IN	NPAC	SPAC
**MED**	**0.003**	<0.001*	<0.001*	<0.001*	<0.001*	<0.001*
**NATL**	0.035*	**0.004**	0.783	<0.001*	<0.001*	<0.001*
**SATL**	0.033*	0.001	**0.000**	<0.001*	<0.001*	<0.001*
**IN**	0.038*	0.010*	0.008*	**0.002**	0.125	0.038*
**NPAC**	0.049*	0.025*	0.022*	0.002	**-**	0.405
**SPAC**	0.051*	0.026*	0.023*	0.003*	0.001	**0.004**

Stock abbreviations: MED (Mediterranean), NATL (North Atlantic), SATL (South Atlantic), IN (Indian), NPAC (North Pacific) and SPAC (South Pacific). F_ST_ values among locations within stocks are shown on the diagonal, and none of them were significant (*p*-value > 0.05).

* significant *p*-value (<0.001)

**Table 3:** Probability of detecting a particular level of differentiation (F_ST_) among populations.

F_ST_	Pχ ^2^
0.0005	0.351
0.0015	0.952
**0.0020**	**0.999**
0.0025	1.000
0.0050	1.000

Regarding adaptation of the populations to the specific environmental conditions of their surroundings, the 17 markers identified as outliers using BAYESCAN were analyzed. The defined haplotype ss974292126+ss974292127 had a positive alpha value and significant high F_ST_ value, suggesting that it may be subject to divergent selection [60]. This haplotype was practically monomorphic in non-Mediterranean samples (fCC = 0.999 and fCA = 0.001), while haplotype frequencies in the Mediterranean Sea were significantly different (fTA = 0.127; fCC = 0.819; fTC = 0.016; fCA = 0.038; F_ST_ = 0.311; p < 0.0001). The remaining 16 outlier SNPs showed a negative value for alpha and significant low F_ST_ values, a result consistent with balancing selection (S3 Fig). Candidate genes involved in essential metabolic pathways were found by homology between our sequence data surrounding the 16 outlier SNPs, and previously known teleost genes (S2 Table).

### Effective population size

**Fig 5:**
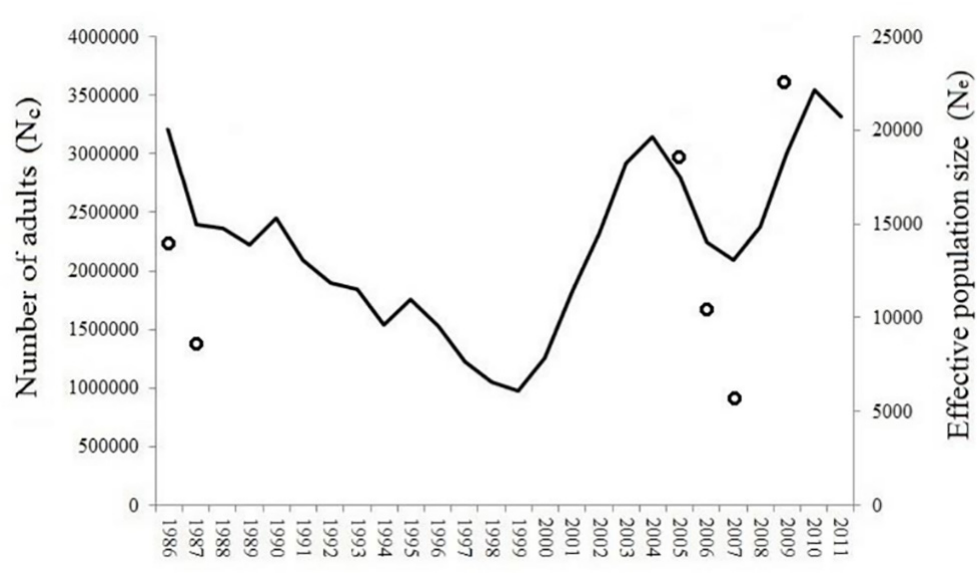
Estimates of the evolution of the number of adults from 1986 to 2011 (solid line, N_c_), and the *short-term* effective population size of each cohort in the North Atlantic (dots, N_e_).

Effective population size (N_e_) was estimated for the North Atlantic Ocean analyzing the 58 neutral SNP markers. While *short-term* N_e_ ranged between 5,466 and 23,330 (C07 and C08 cohorts, respectively) (mean *short-term* N_e_ = 13,267 ± 6,049; S3 Table), *long‐term* N_e_ varied between 13,897 and 20,304 (C08 and C06 cohorts, respectively) (mean *long‐term* N_e_ = 16,729 ± 2,248;S4 Table). Mean *short-* and *long-term* N_e_ were not significantly different (Mann-Whitney U, *p*-value > 0.05). The *short-term* N_e_ was compared to N_c_ (Fig 5), and N_e_/N_c_ ratio (ratio of effective-to-census size) values were found to range between 2.62 × 10^–3^ and 9.83× 10^–3^ (C07 and C08 cohorts, respectively). Despite the apparent correlation between N_e_ and N_c_, it was not found to be statistically significant (r = 0.383; *p*-value = 0.453).

## Discussion

A sustainable management of fisheries requires the exploitation of one single population per stock, and accurate population size estimates [41]. In this way, two problems that reduce intraspecific diversity are avoided: overexploitation and the risk of losing minority populations when various populations are managed as a single stock. The present study is the most comprehensive genetic study carried out to date of the albacore species worldwide. Overall, 117 novel nuclear SNPs were applied to 1,331 albacore individuals from 26 locations covering the whole distribution area of the species. We have described the genetic structure of the species, provided effective population size estimates for the North Atlantic Ocean population, and reported putative signs of natural selection in the albacore genome. Results obtained indicated that none of the currently defined 6 management units includes more than one genetic population. Regarding population size, Ne estimates ruled out the occurrence of severe historical bottlenecks in the North Atlantic Ocean population, and showed that current levels of genetic diversity are sustainable over the time, thereby corroborating the resiliency and responsiveness of the albacore. All these results on albacore population genetic characteristics should contribute to more rational and sustainable fisheries management policies and programs for this important fish species.

### Cross-species amplification of SNPs

This study has shown that cross-species amplification is a valuable approach to identify SNP markers in the albacore species, with a final validation rate of 24.61%. The reciprocal cross was reported by Albaina et al. [34], who showed that albacore and the ABFT species shared 18% of SNPs. Cross-amplification success between Atlantic herring (*Clupea harengus*) and Pacific herring (*Clupea pallasii*) is even lower, 12% [74]. The higher success obtained in the present study lies in the high number of individuals and the assortment of their geographical origins. Here, 1,331 albacore individuals worldwide were studied, whereas four Pacific herrings were analyzed by Helyar et al. [74] and 107 Atlantic bluefin tunas by Albaina et al. [34]. Cross-amplification success also relies on the design of an appropriate SNP set, such as the 384 SNPs from coding regions analyzed in this study. In conserved regions of the genome, such as coding regions, the similarity between the analyzed sequences of two species is increased and therefore, the chance to share SNPs also increases.

Cross-species amplification is considered a valuable approach to identify SNP markers in non-model organisms. Additionally, when these SNPs are located in genes, as is the case in the present study, they can be used for local adaptation studies. In this regard, ss974292126+ss974292127 SNP haplotype was identified as an outlier in BAYESCAN analysis, being highly polymorphic in the Mediterranean while nearly fixed in the three oceans. The result obtained for the ss974292126+ ss974292127 SNP haplotype in the present study is indicative of a clear pattern of diversifying selection. From an adaptive point of view, this result depicts an environmental scenario in the Mediterranean different with respect to the environmental homogeneity of the three oceans. Unfortunately, no homology was found to known teleost’s genes (S2 Table). We also found 16 outlier SNPs with an anomalous homogeneity within the species. One likely scenario is balancing selection actively maintaining those SNPs in the gene pool of albacore. In fact, the inspection of the sequences surrounding the 16 outlier SNPs revealed 13 candidate genes involved in essential metabolic pathways. However, alternative explanations cannot be ruled out: such outliers could also reflect reduced variation at these loci if the minor allele was rare in all populations around the globe, or they could even be false positives. The fast growing genomic data base in the marine world will help to decipher these findings in the near future.

### Population genetic structure

The genetic structure revealed in the present study is reliable since it was based on quite a large sample size, and on an extensive spatial and temporal distribution of samples. Moreover, POWSIM simulations showed that the 50 SNPs with the highest F_ST_ values together yielded a type II error rate (failure to detect a real difference) of 0% for divergences of F_ST_ = 0.002 or greater. In all, 4 spatial-temporally homogeneous populations were identified for the albacore species: Mediterranean Sea, Atlantic Ocean, Indian Ocean, and Pacific Ocean populations (Table 2). When STRUCTURE analysis was repeated for each of the major clusters (Mediterranean, Pacific and Atlantic), no structure was detected within them. This result bears out the findings of Albaina et al. [34]. On the contrary, the genetic heterogeneity within the Mediterranean suggested by others using microsatellite markers [32,33], the observation of separate spawning grounds [39], or differences in isotopic composition [75], was not detected using SNPs. Within the Mediterranean no significant correlation was found between genetic and geographic distances. Present findings also contradicted previously suggested heterogeneity for the Pacific Ocean using microsatellite markers [29,33] or studying migrations, spawning areas and seasons as criteria [76]. And lastly, the Atlantic Ocean was found to be homogeneous in terms of genetic structure, thus challenging earlier results and interpretations on the basis of blood groups [7], microsatellites [29,32] and migratory features [6,77]. In fact, albacore tuna tagging experiments are very scarce, specially in the South Atlantic, Indian Ocean and throughout the Mediterranean Sea. Thus, there is little information about their migratory behavior and the data available are not very informative about population structure and mixing. Moreover, unfortunately, there is little knowledge about albacore spawning areas and times for the different populations [15], and this makes it difficult to get reference samples of known origin (e.g. larvae or young of the year) for genetic studies. Using samples that might represent transient migrants could, in principle, provide a misleading picture of population structure (e.g. suggesting homogeneity within the Mediterranean, where some structure might exist). This potential problem affects mostly at small scales and less at the scale of ocean basins and/or hemispheres. New knowledge about albacore spawning areas and seasons, as well as increased access to reference samples will allow to design more robust genetic experiments to reveal population structure and mixing at smaller scales.

With respect to the discrepancies between the present study and those using microsatellites, and in regard to the power of the markers, although an individual SNP show less power than do multi-allelic microsatellite loci [78], 4–12 nuclear SNPs are expected to have the same power as a single microsatellite locus [79]. Moreover, SNP markers have advantages over other markers: the use of single-tube multiplex assays with small PCR products (60–80 bp) could potentially produce better quality data more efficiently than would genotyping multiple microsatellites, and using SNP loci lies in a more representative sample of the entire genome and a reduced interlocus sampling variance [80].

In this study, neutral SNP variation (Table 2, Figs 3 and 4) and the SNP haplotype putatively under selection showed the Mediterranean group as the most differentiated from the rest of populations. Similarly, extremely different frequencies for *G6PD* locus and mtDNA D-Loop sequences between Mediterranean and Atlantic samples were described by Nakadate et al. [31], and interpreted as indicative of a restricted gene flow. Our results agreed with this, since F_ST_ values between Mediterranean and the rest of populations are the highest, ranging between 0.033 and 0.051 (Table 2). The isolation of the Mediterranean population contrasted with the higher gene flow that occurs between the three Oceans. In fact, when the three Oceans were analyzed together, correlation between genetic and geographic distances of the different sampling points were found (r = 0.7549; *p*-value < 0.0001; S1B Fig), as previously described for the Atlantic herring, another migratory pelagic marine fish [81]. Although there is great evidence that other species (such as Atlantic bluefin tuna and swordfish (*Xiphias gladius*) [82,83,84]) migrate substantially across Strait of Gibraltar, migration is negligible for albacore [6,31]. Results obtained in the present study, together with those using different methodologies, such as genetic markers [30–34], growth parameters [85] and tagging experiments [6] confirm the singularity of the Mediterranean albacore. It is difficult to evaluate whether this singularity is due only to current restricted gene flow, or it may reflect also the demographic history of Mediterranean albacore. According to Kettle et al., [86] the Mediterranean would have served as a refugia for a range of marine species during the last glacial maximum (LGM). Under this latter hypothesis, Mediterranean population would be the result of one major founding event, and would have been isolated from all other populations for a long time. A similar scenario has been proposed for Atlantic herring in the Baltic Sea [87]. In order to shed light on the controversial genetic relationship of the Indian albacore population with that of the Atlantic or Pacific, we analyzed an ample sample including 774 individuals from 12 Atlantic locations, 167 from three Pacific locations, and 136 individuals from four localities in the Western Indian Ocean. Results indicated that Indian samples appeared genetically closer to North Pacific ones, since the FST value between these populations was the only no significant comparison (Table 2). Our work thus confirms with a large sample of the Indian albacore population the results of Albaina et al. [34], who analyzed, also with SNPs, 24 individuals. This sample was the same as that used in the study with microsatellites by Montes et al. [33], although different results were obtained in both studies, since the analysis with 8 microsatellite markers indicated that the Indian albacore population was closer to the Atlantic than to the Pacific one. We think that in this case results may be biased due to the analysis of highly polymorphic markers in a small sample. In any case, the present study also detected that Indian albacore showed both Atlantic and Pacific components in STRUCTURE (Fig 4) and GENELAND analyses. That is, Cape of Good Hope did not represent a definitive barrier to gene flow, as it has been described by other authors [6,28,33].

### Effective population size

Albacore is an overexploited species, whose biomass started decreasing due to overfishing 3 decades ago. Tuna stock assessments based on fishery data are highly uncertain (see [88]) and albacore is not an exception [40,89]. Albacore is a species with seemingly large populations, however they could be more sensitive to genetic drift and inbreeding from intensive harvests than census sizes would suggest [90,91]. In these cases, management requires the maintenance of a much larger census size than would typically be recommended on the basis of information about population dynamics [91]. This is an assumed problem associated to overfished populations: that the high fishing pressure leads to genetic bottlenecks [92,93]. If true, this could have serious implications for management procedures [90,91,94]. Therefore, estimating of N_e_ for sustainable management purposes is a good choice, because it integrates genetic effects with the life history of the species, allowing for predictions of a population's current and future viability [91]. Our analyses on population genetic structure showed no statistically significant spatial or temporal fluctuations within each of the four defined populations. This result indicated that (1) migration had failed to alter allele frequencies at each region, and that (2) the effective population size in each region was large enough to prevent microdifferentiation processes driven by genetic drift. This latter hypothesis was supported for the North Atlantic Ocean population; similar *short-* and *long-term* N_e_ estimates for this population suggested that in spite of the fishing impact on biomass (N_c_), genetic diversity remains high and, therefore, viability of the population has not been affected, this is, it has not suffered severe historical bottlenecks.

From a fishery management perspective, *short-term* N_e_ estimates could provide an approach for generating a fishery-independent indicator of population status. Temporal variations in such an indicator could serve as a prognostic marker of the genetic diversity of exploited albacore tunas and trigger specific well planned management responses to signs of reduced diversity (e.g. drastic reduction of fishing effort until genetic diversity is recovered). Management must often default to apparently simple rules-of-thumb, such as the 50/500 criteria for maintenance of genetic diversity; this means that a *short-term* N_e_ ≥ 50 is required to avoid the damaging effects of inbreeding, and a *short-term* N_e_ ≥ 500 is necessary to avoid extinctions due to the inability to evolve to cope with environmental change. Taking this rule into account, we have demonstrated that albacore population size in the North Atlantic Ocean is high enough for dealing with both, inbreeding effects and adaptation capabilities. But for management purposes, N_e_ estimates might be more adequate to better understand how ecological factors reduce or increase the N_e_/N_c_ ratio. With this regard, theory suggests that N_e_/N_c_ ratios in the wild should be above 0.1 [95–97], and empirical evidence for several wild populations of different non marine species is consistent with this prediction, showing N_e_/N_c_ ratios ranging from 0.10 to 0.14 [96,98]. In the North Atlantic Ocean, the effective population size was three orders of magnitude lower than the adult census size (S3 Table). These figures are within the range documented for other fish species, such as *Sciaenops ocellatus* [99], *Pagrus auratus* [92] and *Sebastes crameri* [100]. A low N_e_/N_c_ ratio could be explained by variance in albacore survival due to high larval and pre-recruit mortality [101,102], indicating that few mature adults contribute to each generation. It has been questioned the appropriateness of estimating N_e_ from temporal data in species with high effective population sizes [90], and whether N_e_/N_c_ ratios reflect the true dynamics of biological systems [94,103]. Nevertheless, it is important to obtain a better understanding of how vulnerable fish populations are to loss of genetic variation and in that respect, the data presented here on temporal stability at neutral markers will serve as an important baseline for future evaluations of N_e_/N_c_ and for monitoring N_e_ in albacore. In conclusion, N_e_ estimate, as a fishery-independent index of abundance, provides a valuable complementary tool for monitoring the status of fish populations in order to implement more sustainable management actions.

## Supporting Information

S1 Fig Isolation by distance. a) Regression of pairwise geographic distance and genetic similarity for the 26 locations. b) Similar analysis using 19 locations from the Atlantic, Indian and Pacific Oceans and c) considering only the 7 Mediterranean samples.
(TIFF)

S2 Fig Estimated number of populations from STRUCTURE (a) and GENELAND (b) analyses. (a) Mean probabilities of the data [LnPr(X)|*K*] over 10 STRUCTURE replicated runs plotted as a function of putative number of clusters (*K*). (b) Posterior density distribution of the number of estimated clusters.
(TIFF)

S3 Fig BAYESCAN output plot for outlier identification. Graphical representation of the markers based on F_ST_ values (y axis) against log(*q*-value) (x axis). Candidate markers under selection are those with a *q-*value less than 0.1, represented at the right of the vertical black line. Grey circles represent candidate markers for balancing selection and black circles represent candidate marker for divergent selection; empty circles represent putatively neutral loci.
(TIFF)

S1 Table Characteristics of the 117 genotyped SNPs in 1,331 *Thunnus alalunga* samples analyzed in this study. (*) SNPs obtained from Albaina et al. [34].
(TIFF)

S2 Table BLASTn results and possible molecular function of eighteen sequences of candidate SNPs under divergent or balancing selection. (TIFF)

S3 Table *Short-term* N_e_ estimate for the North Atlantic from 1986 to 2009 based on the temporal method. Generation time (Ĝ), F_s_ values, harmonic means of effective (Ň_e_), spawning census population size (Ň_c_), and Ň_e_/Ň_c_ ratio.
(TIFF)

S4 Table *Long-term* N_e_ estimate for the North Atlantic based on the coalescent method. Number of samples representing each cohort (N), spawning census population size (N_c_) and N_e_/N_c_ ratio values are listed.
(TIFF)
