# Supplementary figures and images for: New Nuclear SNP Markers Unravel the Genetic Structure and Effective Population Size of Albacore Tuna (Thunnus alalunga)
Source: PLoS One. 2015 Jun 19;10(6):e0128247. doi: 10.1371/journal.pone.0128247 (PMC4474438; doi:10.1371/journal.pone.0128247)

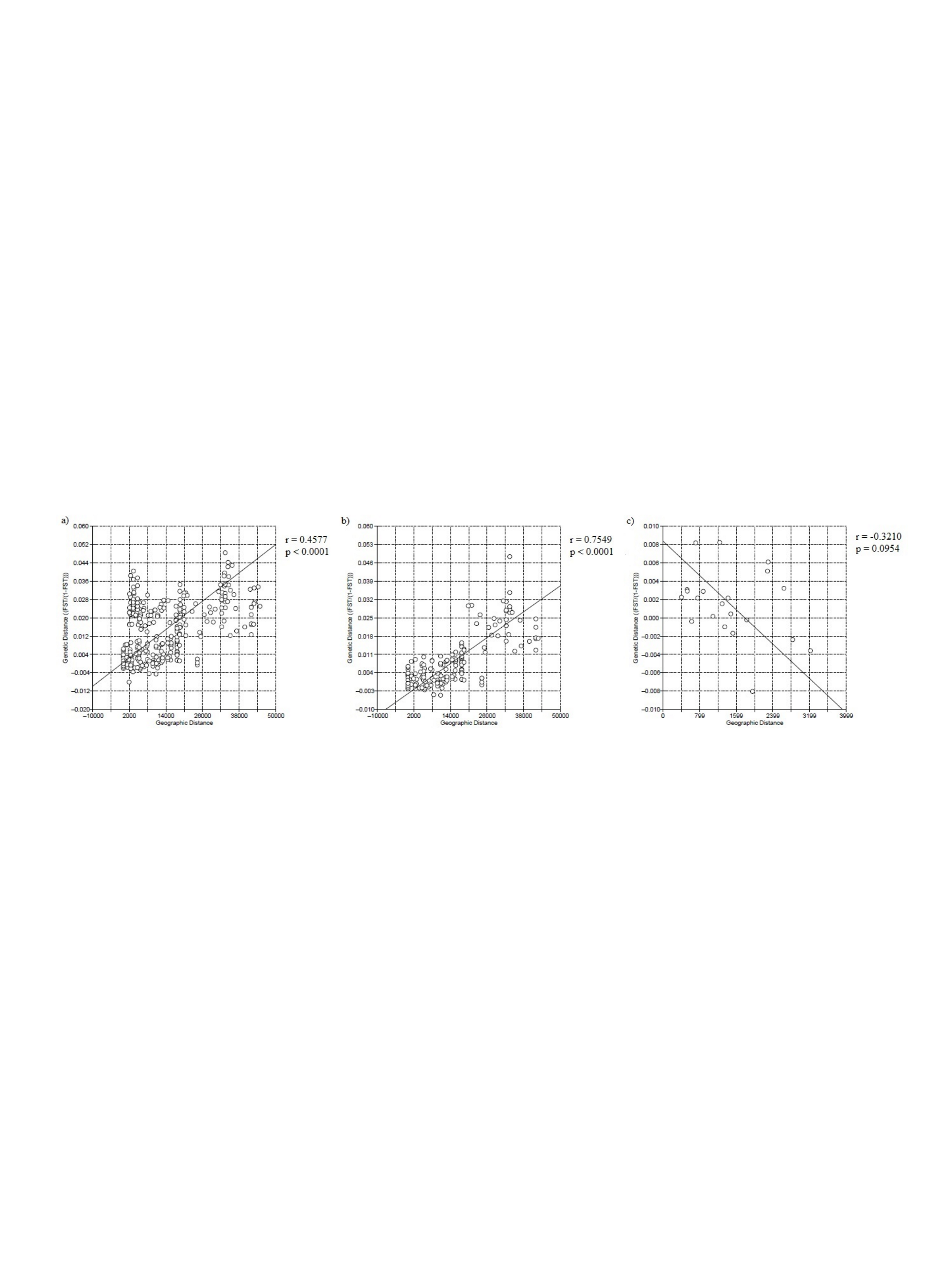

Supplement: S1 Fig — a) Regression of pairwise geographic distance and genetic similarity for the 26 locations. b) Similar analysis using 19 locations from the Atlantic, Indian and Pacific Oceans and c) considering only the 7 Mediterranean samples. (TIFF) [file pone.0128247.s001.tiff]

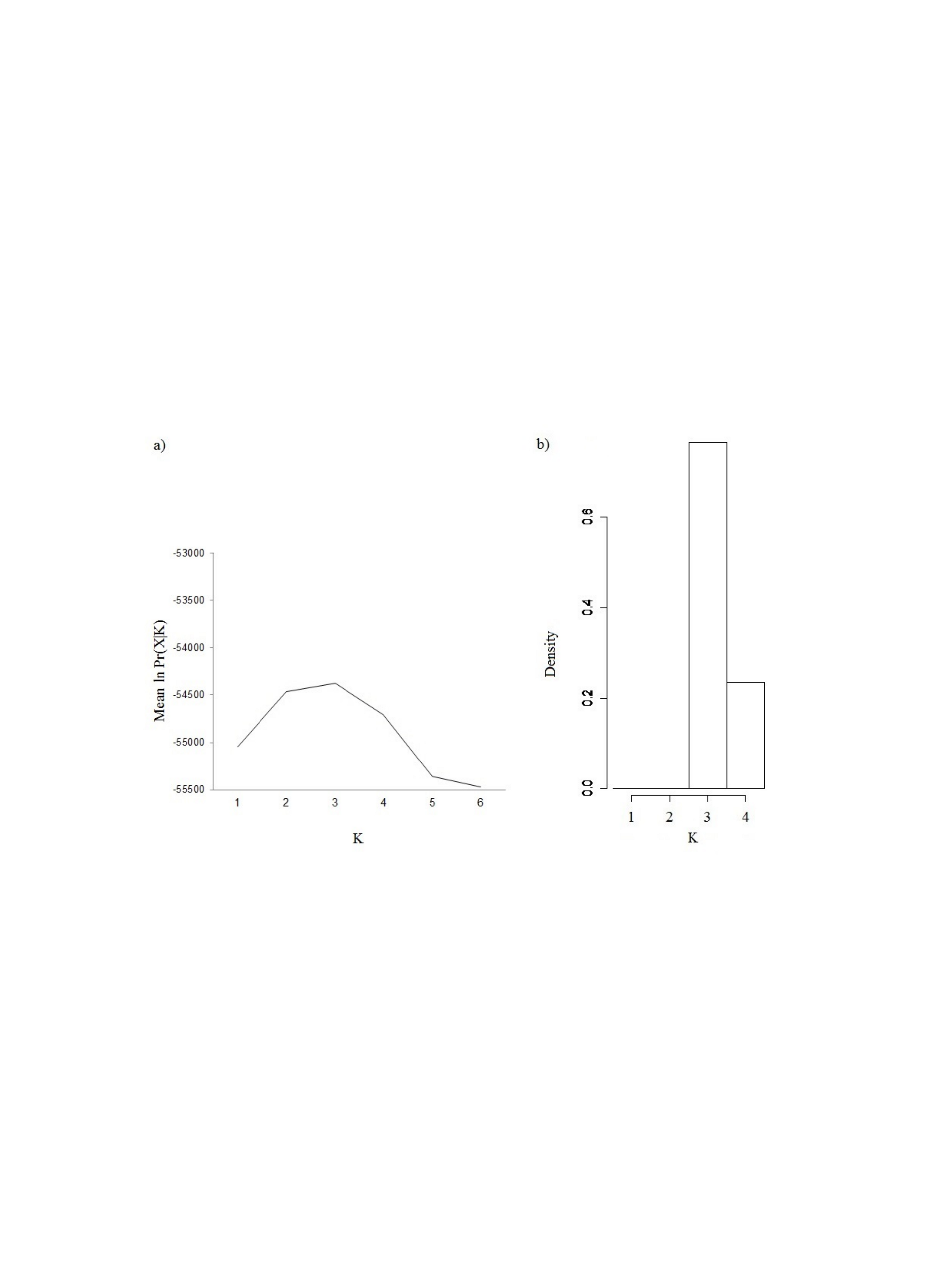

Supplement: S2 Fig — (a) Mean probabilities of the data [LnPr(X)|K] over 10 STRUCTURE replicated runs plotted as a function of putative number of clusters (K). (b) Posterior density distribution of the number of estimated clusters. (TIFF) [file pone.0128247.s002.tiff]

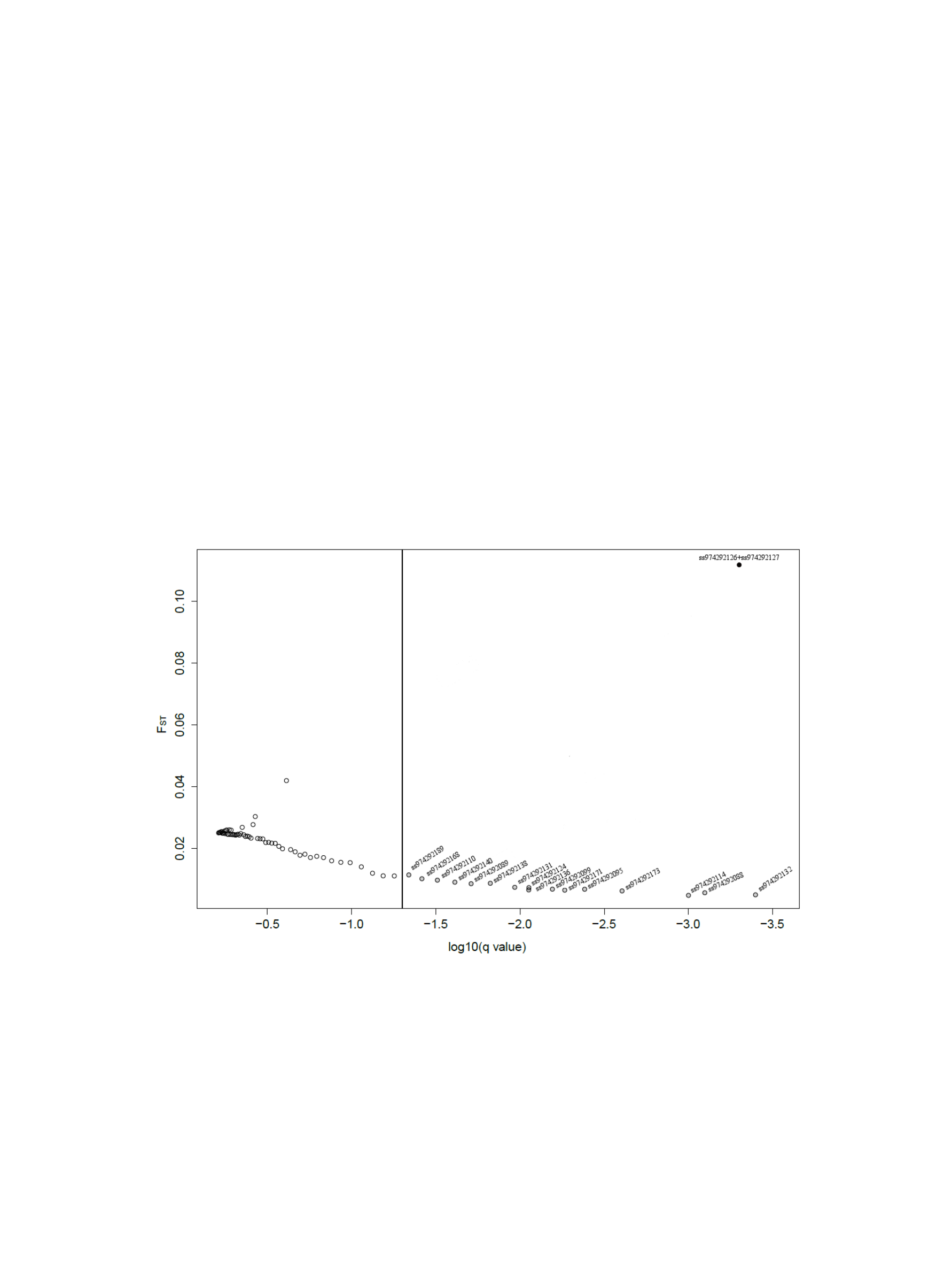

Supplement: S3 Fig — Graphical representation of the markers based on FST values (y axis) against log(q-value) (x axis). Candidate markers under selection are those with a q-value less than 0.1, represented at the right of the vertical black line. Grey circles represent candidate markers for balancing selection and black circles represent candidate marker for divergent selection; empty circles represent putatively neutral loci. (TIFF) [file pone.0128247.s003.tiff]

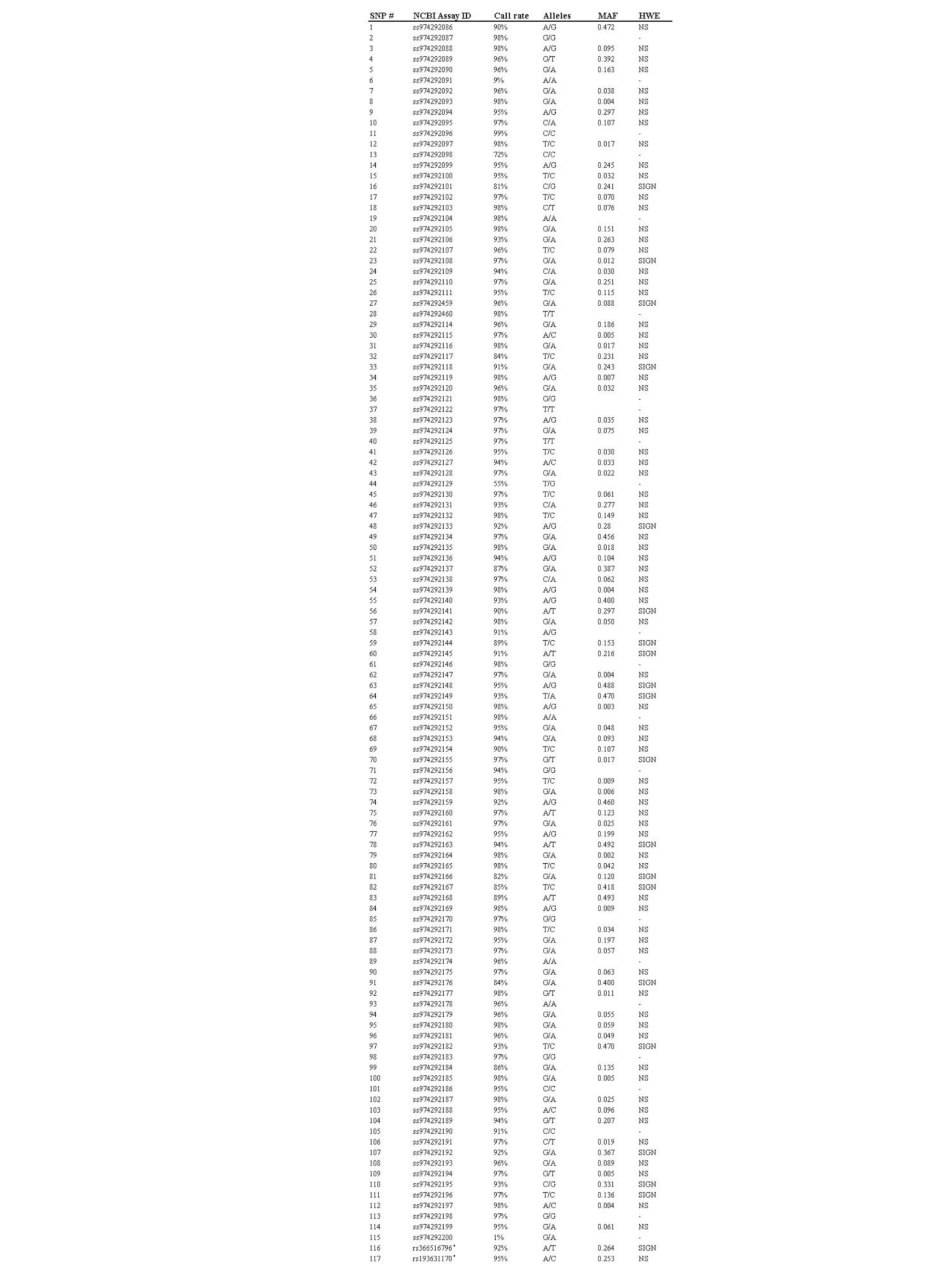

Supplement: S1 Table — (*) SNPs obtained from Albaina et al. [34]. (TIFF) [file pone.0128247.s004.tiff]

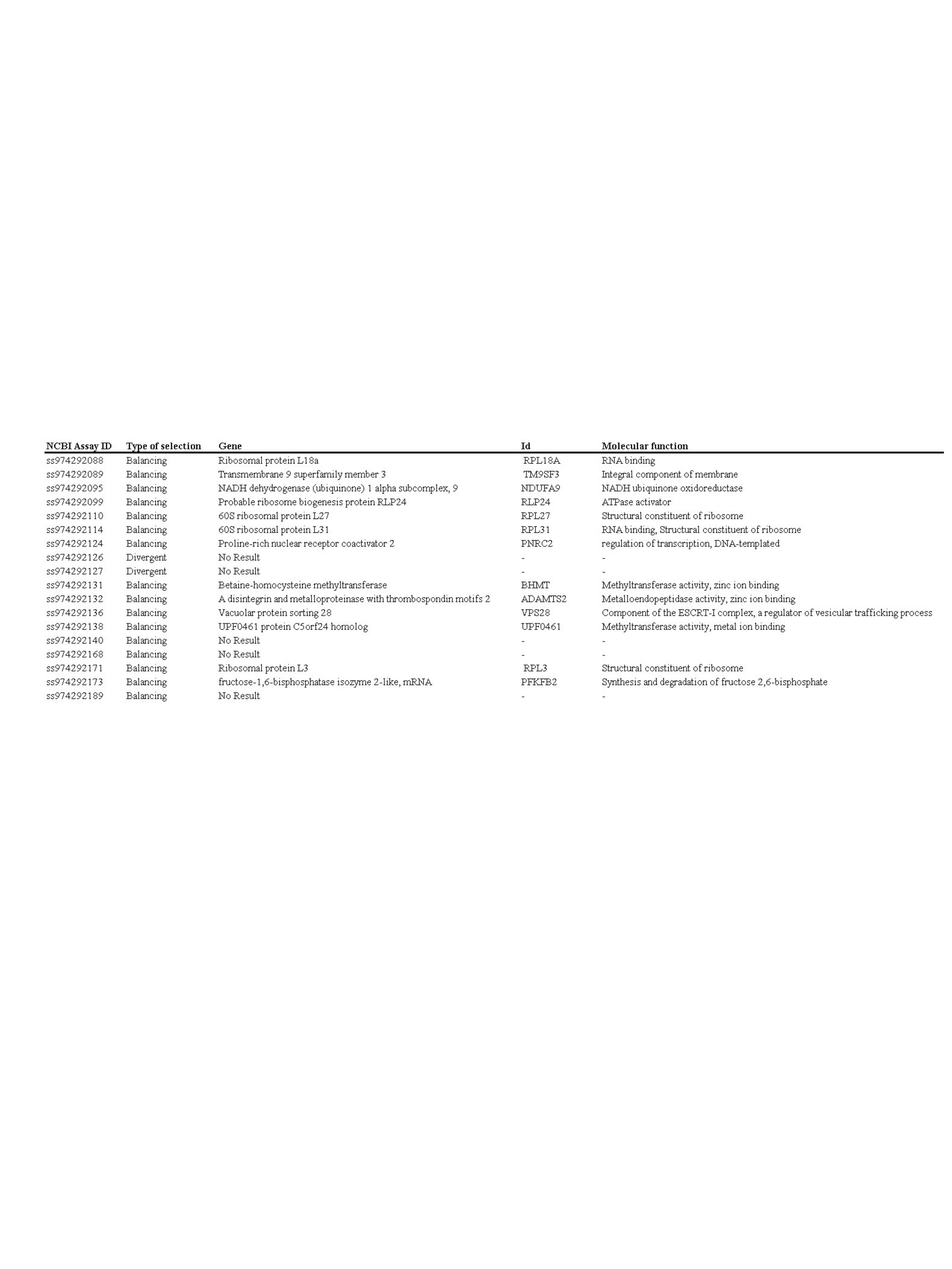

Supplement: S2 Table — (TIFF) [file pone.0128247.s005.tiff]

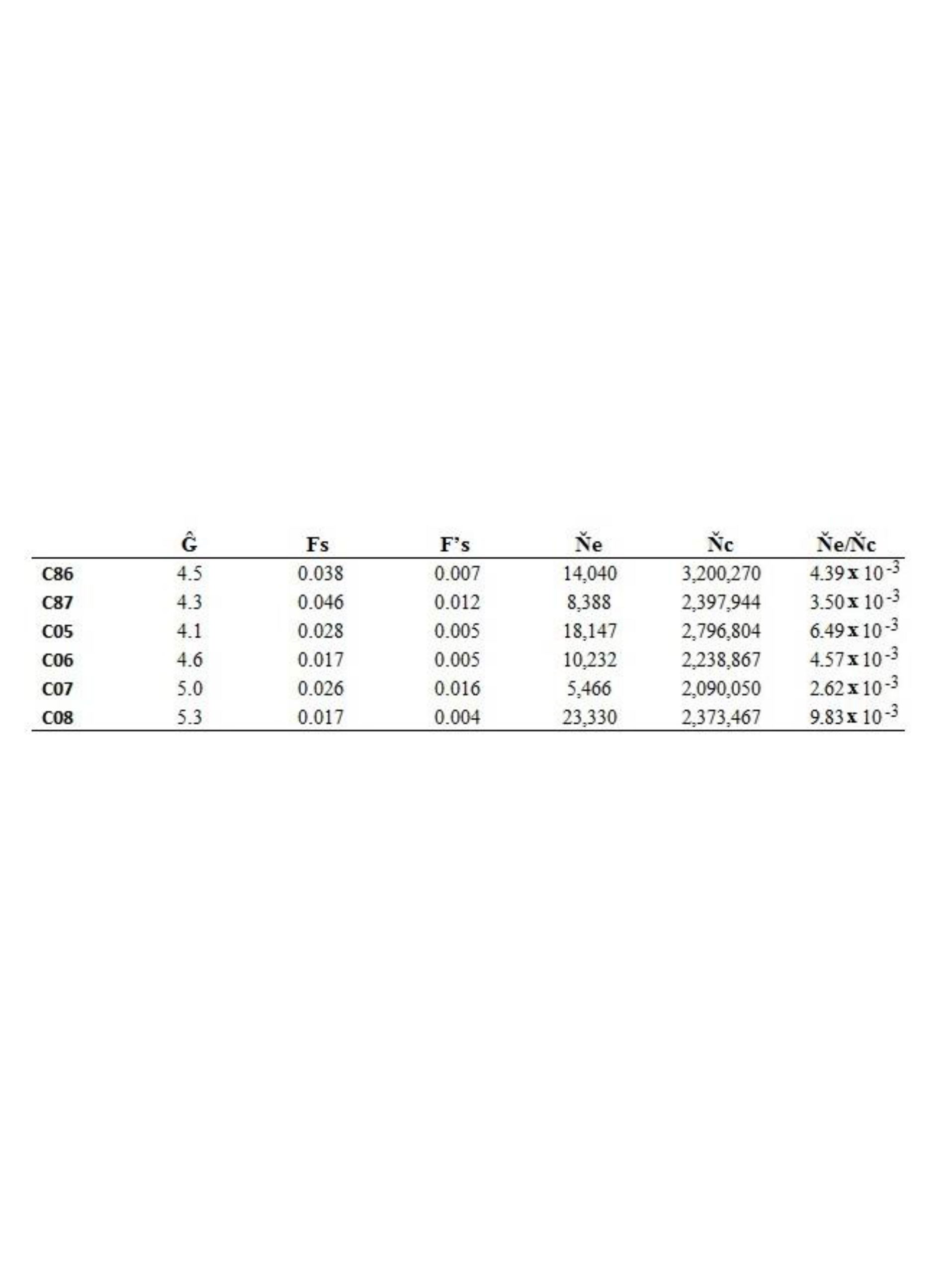

Supplement: S3 Table — Generation time (Ĝ), Fs values, harmonic means of effective (Ňe), spawning census population size (Ňc), and Ňe/Ňc ratio. (TIFF) [file pone.0128247.s006.tiff]

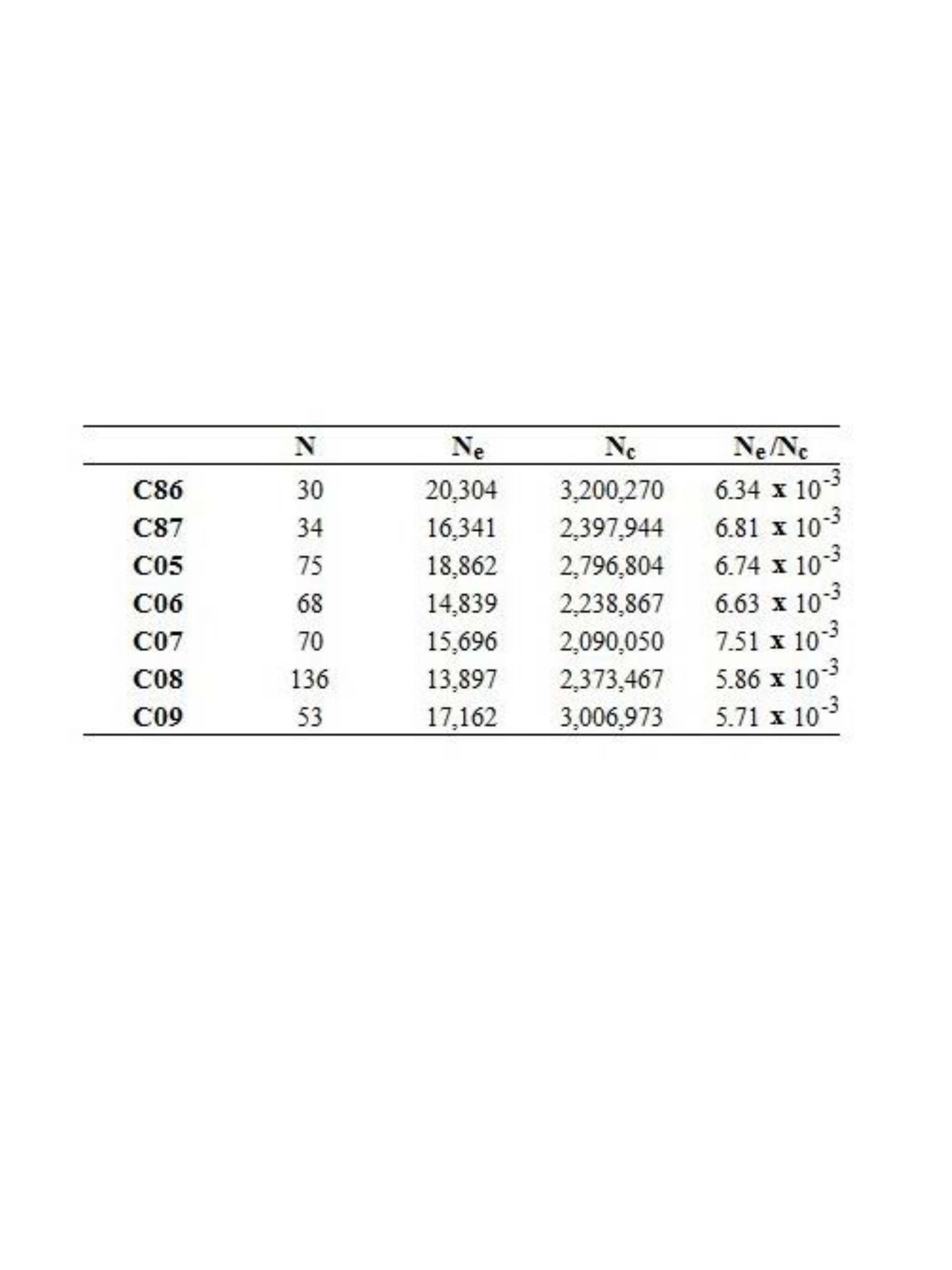

Supplement: S4 Table — Number of samples representing each cohort (N), spawning census population size (Nc) and Ne/Nc ratio values are listed. (TIFF) [file pone.0128247.s007.tiff]
